# Semi-Automatic Tool for Vitiligo Detection and Analysis

**DOI:** 10.3390/jimaging6030014

**Published:** 2020-03-24

**Authors:** Paolo Neri, Michela Fiaschi, Giovanni Menchini

**Affiliations:** 1 Department of Civil and Industrial Engineering, University of Pisa, 56122 Pisa, Italy; 2 Dermacademy Institute for Dermatological Sciences and Aesthetic Medicine, 56121 Pisa, Italy

**Keywords:** vitiligo, image processing, semi-automatic vitiligo detection, black light

## Abstract

Vitiligo vulgaris is an autoimmune disease which causes a strong reduction of the cells producing melanin, which is the main skin pigment. This results in the growth of white patches on patients’ skin, which are more or less visible, depending on the skin phototype. Precise, objective and fast detection of vitiligo patches would be crucial to produce statistically relevant data on huge populations, thus giving an insight on the disease. However, few methods are available in literature. In the present paper, a semi-automatic tool based on image processing to detect facial vitiligo patches is described. The tool requires pictures to be captured under black light illumination, which enhances patches contrast with respect to healthy skin. The user is only required to roughly define the regions of interest and set a global threshold, thus, no specific image-processing skills are required. An adaptive algorithm then automatically discerns between vitiligo and healthy skin pixels. The tools also allow for a statistical data interpretation by overlapping the detected patches of all patients on a face template through an occurrence map. Preliminary results obtained on a small population of 15 patients allowed us to assess the tool’s performance. Patch detection was checked by an experienced dermatologist, who confirmed the detection for all the studied patients, thus supporting the effectiveness of the proposed tool.

## 1. Introduction

Vitiligo vulgaris is an autoimmune disease with an unpredictable progress: it generally shows a slowly progressive course, but spontaneous remissions are also possible. The population incidence worldwide is 1%, without significant associations with race or sex. Currently, no prognostic index exists, and no standard symptomatic reference scheme has been established to evaluate the clinical course of the disease, which would help dermatologists to choose an appropriate therapeutic path and guide researchers in approaching new treatments. Vitiligo is characterized by the lowering of the cells that produce the main skin pigment, melanin [1,2]. The patches of vitiligo can occur in any body area and in any size, even covering the entire skin surface. In dark-phototype people, vitiligo is easily visible and induces socio-relational problems. In clear-phototype individuals, vitiligo is instead generally less visible. Due to this disparity of visibility, ultraviolet illumination (black light) may be helpful to correctly study this disease in each skin phototype, since it enhances the natural fluorescence of the epidermis, which is generally masked by the presence of melanin.

A precise detection of patch location on patients’ bodies, performed on a large population, represents the first step for a reliable statistical analysis of the phenomenon. Indeed, this analysis, correlated with other parameters such as onset age and associated diseases, may help the researchers to define and categorize the different variants of vitiligo, since a rigorous clinical classification is still missing [3,4,5]. Also, the patches detection and area measurement can help assessing the treatment effectiveness [6,7]. To this purpose, recent studies demonstrated that black light (around 365 nm [8]) is crucial in highlighting vitiligo patches, enhancing the contrast with respect to healthy skin [9]. In particular, in subjects with a clear-skin phototype it is very difficult, if not impossible, to see the difference between healthy skin and vitiligo patches without black light illumination. Even if black light illumination is not mandatory for dark-skin phototype populations, it can still be needed for areas with little melanin such as the palms of the hands and soles of the feet. Thus, the examination with black light is considered the gold standard in the evaluation of this disease, and it is a common practice by most dermatologists [10].

In last years, the international scientific literature has been starting to outline the possibility that there are two forms of bilateral vulgar vitiligo, showing some peculiarities. The first, and most documented in literature, is the onset age [11]: one form has onset in childhood [12], while the other form has onset in adulthood [5]. However, there are no known clinical differences which allow to definitively characterize a form with respect to the other [3,4,5,12]. There are other differences between these two forms that emerge from clinical practice, but they were not rigorously studied yet nor documented in literature. A possible characteristic which identifies the two forms could be the site of the onset of vitiligo patches on the face, which is an ideal location for studies since it has numerous skin areas in which the patches can arise. Even if further investigation would be needed on this topic, there are no methods in literature that allow to rigorously identify and stratify the presence of vitiligo in the various face areas [13]. Fully manual image elaboration was presented to detect and measure vitiligo patch area [14]. Also, some papers describe the use of computer-aided image processing tools for vitiligo patch detection, but the papers are more focused on the clinical aspects and they do not provide a deep insight on the method itself [15,16]. Also, these methods are based on the presence of green markers on a patient’s skin. This represents a severe limitation, since a marker for each patch is needed, thus small patched area may be missed. Also, the method cannot be applied to existent database acquired without the markers. Finally, the use of machine learning techniques show promising results in the last years [17]. These approaches require detecting vitiligo patches in a set of training images with other techniques (often manually), then allow using the trained neural network to automatically process a larger data set. It is worth noting that this approach is not feasible for small data sets. Thus, in this paper an image-processing tool is proposed for semi-automatic patch detection, focusing on the patient’s face. The tool accepts a rough definition of patch contours and allows setting an adaptive threshold parameter which is used to automatically discern between healthy and patched skin regions. The tool also allows for the statistical analysis of the studied population. This task is generally manual, by defining some rectangular, numbered face areas and assessing if vitiligo is present or not in that area [18]. This is a cumbersome method unfeasible for large image databases. The method itself is not objective and does not allow to precisely resolve patches that cross different areas. In the present paper, data elaboration is presented, enabling the researcher to display the detected vitiligo regions altogether on a face template, associating each face region with the percentage of patched patients in those regions with a color map.

## 2. Materials and Methods

The proposed approach is based on a semi-automatic tool which exploits image-processing techniques to detect vitiligo patches on a patient’s face. All subjects gave their informed consent for inclusion before they participated in the study. The study was conducted in accordance with the Declaration of Helsinki, and the protocol was approved by the Ethics Committee of “Azienda Ospedaliera Universitaria Policlinico P. Giaccone” (10 April 2013, N.5/2013). The tool was programmed in Matlab language. The software was developed referring to the end users (i.e., dermatologists), who do not necessarily have any experience neither in image-processing or in Matlab coding. Thus, an interactive graphical interface was developed to guide the user through the vitiligo detection. The information of each patient (stored in a Matlab structure) can then be saved in a “*.mat” file for subsequent elaboration.

### 2.1. Graphical Interface

**Figure 1:**
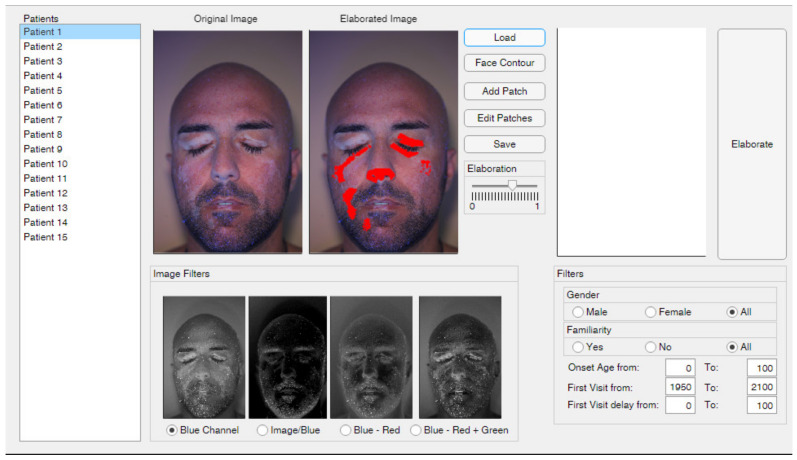
Overview of the graphical interface.

The developed graphical interface allows to perform all image processing operations in a user-friendly environment. Figure 1 shows an overview of the interface, which can be ideally divided in three parts: the patient list is displayed on the left side, the detection tools are shown on the central region and the overall patient results (and filters) are displayed on the right side of the interface.

The *“Load”* button is used to populate the patient list. The software imports patient data from a properly formatted Excel file, which contains patient names and some personal information, such as patient age, date of first visit and date of vitiligo onset. While personal data are crucial for statistical analysis, patient names are needed to associate each patient with the corresponding picture. The patient list is also used to browse among the patients to be elaborated: a click on a patient’s name displays the corresponding image and the previous elaboration results, if available. More precisely, the *“Original Image”* box shows the patient’s color photograph without any elaboration, except a resolution re-scaling. Indeed, the images are generally acquired with high-resolution cameras. Such high resolution may result in a slower elaboration process, while not providing any practical advantage. The resolution can somehow affect the definition of patch edges, causing negligible variations of the patch areas and not altering the patch location with respect to the face. Moreover, the elaboration of images acquired with different resolutions (e.g., acquired in different years with different cameras) may artificially affect the results, since the patches areas are computed based on the pixel count. To overcome these issues, each image was scaled to a pre-defined resolution of 610 × 407 pixels, which represents approximately 0.1 of the native resolution and corresponds to the sizes of the face template used for the statistical elaboration (see Section 2.6). On the other hand, the *“Elaborated Image”* box shows the elaboration results, highlighting with red dots the pixels were vitiligo patches are detected. This plot is updated each time that the elaboration settings are changed, i.e., the threshold value and the selected image filter. The comparison between original and elaborated images allows the dermatologist to assess the effectiveness of the selected elaboration parameters. The analysis of Figure 1, for example, allows assessing that the vitiligo patches on the left eye were not properly detected, thus the elaboration should be refined to obtain reliable results. 

### 2.2. Face Contour

**Figure 2:**
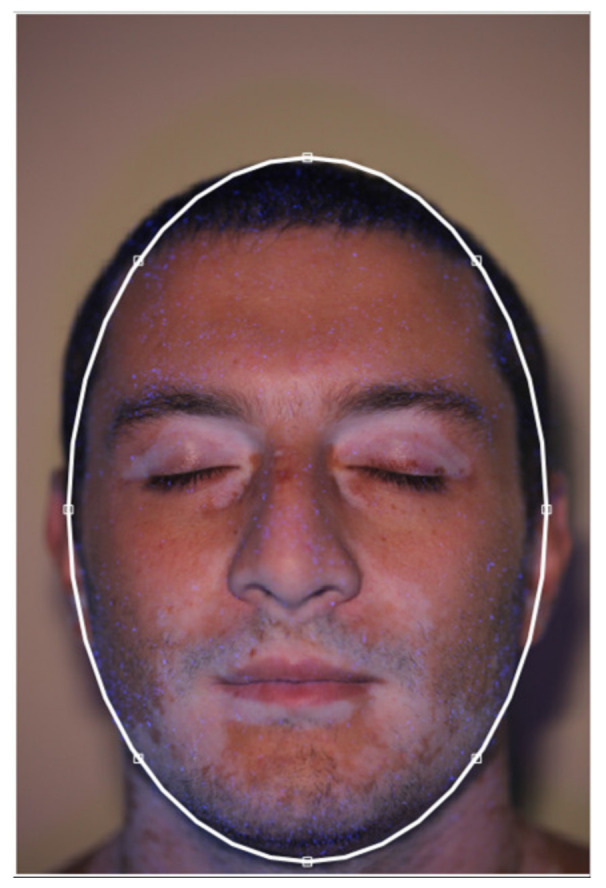
Patient’s face contour definition.

The *“Face Contour”* button allows the user to define the face edges. This is approximately achieved by interactively drawing an ellipse on the original image, as shown in Figure 2, to define the location and the dimensions of each patient’s face. This step is needed to avoid misleading comparisons of pictures imaged with different perspectives or at different distances. The face contour is then used for two different purposes: to compute the area of the patient’s face (which can be compared with the area of the vitiligo patches) and to align the locations detected on each different image with respect to the template face. It is worth noting that the ellipse only represents a rough description of the patient’s face, and more complex geometry (such as splines) could be implemented in future developments. A more accurate description of the face contour will lead to a longer elaboration time, but would not bring substantial benefits, since the actual face location in the image and its main dimensions can be properly described by the ellipse.

### 2.3. Add and Edit Patches

**Figure 3:**
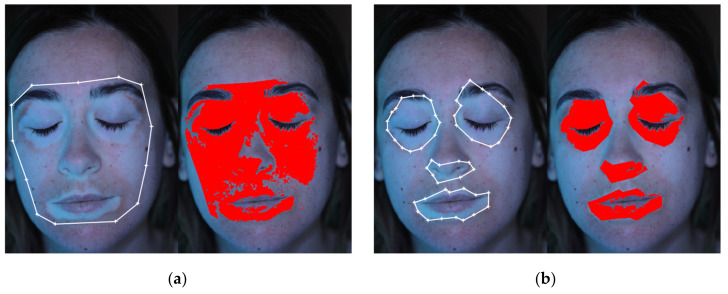
Comparison between different regions of interest (ROIs) definition: (**a**) Single ROI for the whole face; (**b**) Multiple separate ROIs for different patches.

The detection of vitiligo patches is performed based on the contrast between the healthy skin regions and the affected regions. As stated, this contrast is enhanced by the use of black light during image acquisition. The actual color and intensity of each patch depends on several variables, such as natural skin color, illumination directions, face anatomy, residual make-up, etc. Thus, the fully automatic detection of each patch of the patient is quite challenging; the extension of the same algorithm to different patients is nearly impossible. Thus, the definition of a fully automated procedure is not feasible since a relevant contribution of the user would still be needed to check and to correct the automatic results. For these reasons, a semi-automatic procedure was preferred at this research stage. While global considerations of colors and intensity are not reliable, the patch regions are locally easily detectable and highly contrasting. Thus, user input defining the regions of interest (ROIs) containing the patches is enough to constraint an automatic locale detection algorithm and to obtain consistent results. This can be interactively achieved with the *“Add Patch”* and *“Edit Patches”* buttons. The *“Add Patch”* button allows drawing a polygonal region which should represent a rough contouring of the ROI to be added to the analysis. The number of ROIs to be defined depends on the specific patient and on the lighting conditions. Each ROI defines an area in which the patch is to be detected. Since the automatic detection algorithm is based on an image thresholding (see Section 2.5), the defined ROIs are also used to obtain an adaptive threshold value. The white vitiligo patches indeed are characterized by lighter colors and higher intensity level. Thus, the maximum intensity value of each ROI is computed and used to scale the selected global threshold. Thus, better analysis results are obtained if ROIs are defined in regions with even illumination, while shady areas containing vitiligo patches can be reliably analyzed if contoured with dedicated ROIs. A typical example is provided by the eyebrows and eyes regions. While the eyebrows, located on the forehead, are generally highly exposed, the eyelids are generally darker. Thus, if a single ROI is drawn for both the areas, automatic thresholding may fail. On the other hand, if the two portions are analyzed with different ROIs, more reliable results can be achieved. This effect can be assessed by looking at Figure 3. In Figure 3a, a single ROI was defined for the whole face and, as can be noted, it was not possible to separate vitiligo pixels from overexposed chicks’ pixels. On the other hand, in Figure 3b, multiple ROIs were defined for the various vitiligo patches. Even if the global threshold was set to the same value for both Figure 3a,b, the multiple ROIs elaboration shown in Figure 3b leads to much more reliable results, thanks to the adaptivity of the proposed algorithm.

### 2.4. Image Filters

**Figure 4:**
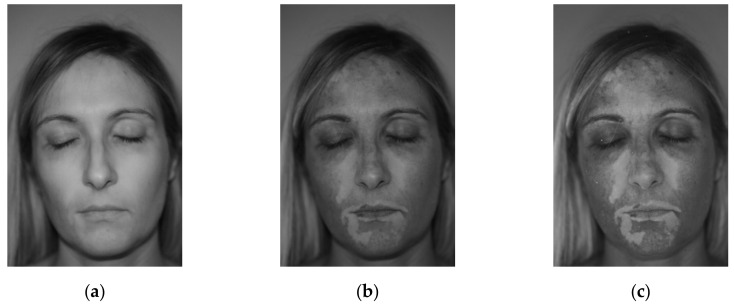
Sample color picture: (**a**) Red channel; (**b**) Green channel; (**c**) Blue channel.

The proposed tool needs color pictures as input images (i.e., 3-channel RGB images), acquired with black light illumination. It is worth noting that the need of black light illumination does not represent a severe limitation of the method, since it represents the common dermatologist practice. Even if patches detection methods based on colors can be developed, they can be more sensible to patients’ specific characteristics, such as skin natural color, tanning, residual make-up, etc. Thus, the proposed approach was based on a gray-level thresholding method. In order to keep the color information, four different image elaborations are proposed, which can be chosen depending on the specific patient. Different filters can be evaluated and selected in the bottom-central part of the graphical interface, as shown in Figure 1. In particular, the use of a black light causes the vitiligo patches to be more contrasted in the blue channel of the image, as highlighted by the comparison between Figure 4a–c, which represent the data of the red, green and blue channels of a sample image respectively.

**Figure 5:**
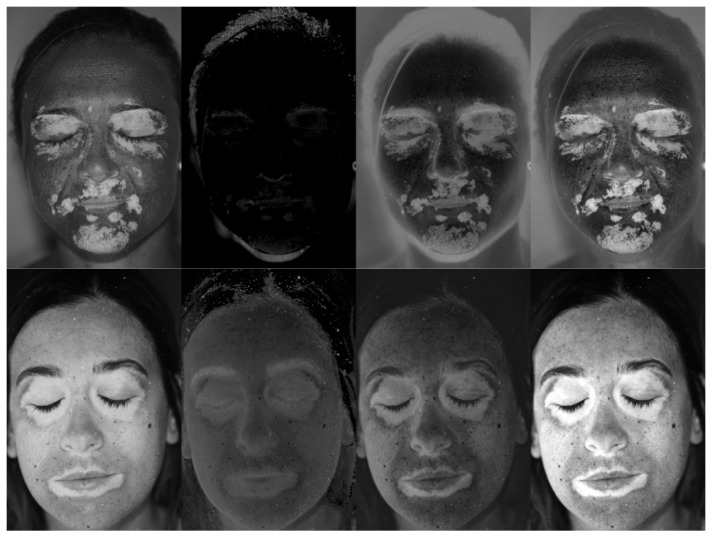
From left to right, filter 1, filter 2, filter 3 and filter 4 applied to the images of Patient 15 (**top**) and Patient 8 (**bottom**).

As can be noted, the red channel (Figure 4a) almost loses any information about the vitiligo patches. The green channel (Figure 4b) shows the patches, but appears darker and less contrasted. The blue channel (Figure 4c) shows the highly contrasted patches, being the brightest image. For this reason, the first available option of the detection tool is to only consider the blue channel for the subsequent automatic detection (filter 1). The second available option allows normalizing the image with respect to the blue channel. More precisely, each of the three channels is divided by the blue channel image in an element-wise fashion. The resulting 3-channel image is then converted to gray scale by exploiting the *rgb2gray* Matlab function. The obtained image has a low gray level in correspondence of high blue-channel values (i.e., vitiligo patches) and vice versa. Thus, the complementary image is computed, in order to represent the vitiligo patches with high gray levels (filter 2). In order to further enhance the image contrast, the third and the fourth available image filtering are represented by gray images obtained by subtracting the red channel, respectively, to the blue channel (filter 3) and to the sum of the green and blue channels (filter 4). Figure 5 shows an example of the described filtering applied to the pictures of Patients 15 and 8. As can be noted, different filtering approaches can be relevant depending on the specific patient. The automatic patch detection is updated each time that a different filter is selected, allowing to evaluate which choice is more suitable for each patient.

### 2.5. Elaboration

The developed tool performs the automatic patch detection algorithm each time the user changes the image filter or the global threshold value (which ranges from 0 to 1, and is set by the user through a slider). The algorithm is divided in several steps. Firstly, a black image with the same dimensions of the original image is initialized. The analysis is then performed on each ROI separately through a *for*-loop. The *i*-th polygonal ROI is selected and used to mask the patent’s filtered image: the values outside the ROI are set to 0, while the values inside the ROIs are saved. Then, the maximum gray level in each ROI is computed. The algorithm exploits the global threshold value to define an adaptive threshold, multiplying the global threshold value by the maximum gray level of each ROI. This allows to scale the threshold with respect to the actual brightness of each ROI. The masked image is then binarized, i.e., all the values lower than the selected adaptive threshold are set to 0, while all the values greater or equal to the threshold are set to 1. The obtained binary image reports in white the pixels detected as vitiligo patches. In practice, if a global threshold equal to 0 is selected, all the pixels of the ROI are detected as vitiligo patches. On the other hand, if a global threshold equal to 1 is selected, only the pixels corresponding to the highest gray level are detected as vitiligo patches. The tuning of the global threshold level, in conjunction with the proper definition of the ROIs and the image filtering, allows for an accurate definition of the vitiligo patches. It is worth noting that, in the case of low contrast or ambiguous patches, it is always possible to manually contour the patch by defining a really fine ROI and to set the global threshold close to 0. This allows obtaining a “manual” patch detection in the case of automatic algorithm failure. Nevertheless, a proper ROI definition and global threshold selection generally results in a successful automatic detection, which is the case of all the 15 images analyzed in this research (see Section 4).

### 2.6. Results Representation

**Figure 6:**
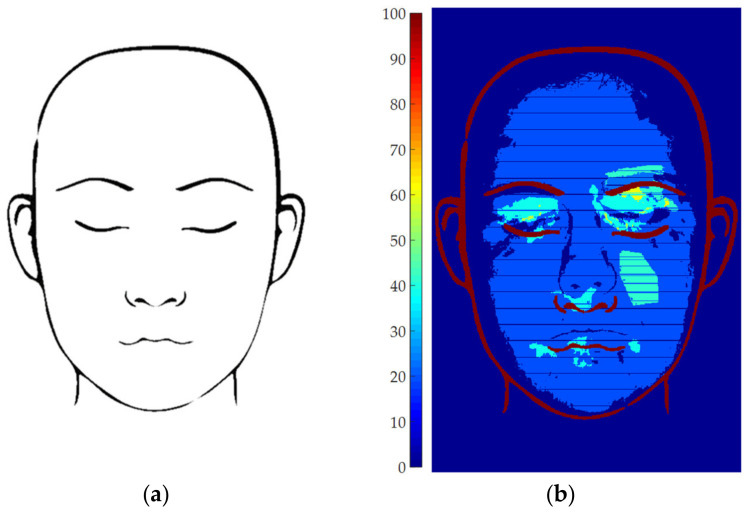
Statistical elaboration: (**a**) Face template; (**b**) Example of occurrence map computed analyzing 5 patients.

The rightmost region of the GUI was used to display the statistical elaboration data. The patient data stored after the processing of each single image may be loaded and elaborated by pressing the *“Elaborate”* button. The aim of this elaboration is to overlap the results extracted from each patient on a single face map which represents the percentage of patients showing vitiligo patches in each region of the face, overlapped to a face template for reference (Figure 6a). Thus, in this map, a value of 100% is obtained in the locations where all the studied patients show vitiligo, and vice versa. To achieve this result, an occurrence matrix ***M*** is initialized as a 610 × 407 zero matrix, corresponding to the dimension of the template face image. Then, an iterative process is setup, processing the whole patient database. At the *i*-th step (*i* = 1...*N*, being *N* the total number of analyzed patients), the coordinates of the pixels showing vitiligo for the *i*-th patient are considered. These coordinates need to be normalized to fit the face template. Both vertical and horizontal scaling factors are computed basing on the face ellipses: the principal axes of the template ellipse are divided with respect to the patient’s ellipse principal axes. Thus, the pixel coordinates are scaled with the obtained scaling factors and then translated so that the center of the patient’s ellipse coincides with the center of the template ellipse. The *round* operator is finally used to obtain only integer scaled pixel coordinates. It is worth noting that this rounding step could cause some artifacts in data interpretation, and it could be replaced with more sophisticated data interpolation. Since the software is intended to be used on a large number of patients, the possible differences at pixel level were considered to be negligible. Then, the matrix ***m****_i_* was defined as a 610 × 407 matrix: the elements of ***m****_i_* corresponding to vitiligo pixels for the *i*-th patient were set to 1, while all the other elements were set to 0. Thus, at each *i*-th step the global occurrence matrix ***M*** is updated as:

***M*** = ***M*** + ***m****_i_* (1)

At the end of the for loop, the summation of the ***m****_i_* matrixes leads to a global occurrence matrix ***M*** whose elements values are equal to the number of patients showing vitiligo at that coordinates. Thus, it is sufficient to normalize ***M*** with respect to the total number of patients to obtain the occurrence percentage. This map can be finally overlapped to the face template and displayed as a color map, as shown in Figure 6b.

It is worth noting that the blue horizontal lines shown in Figure 6b, which apparently corresponds to zero-occurrence straight lines, is an artifact due to the pixel coordinates rounding step. Indeed, the above example was obtained by elaborating 5 images only, which are not statistically relevant. The influence of this phenomenon can be drastically reduced by increasing the number of analyzed patients. Nevertheless, this small set of patients allows to easily distinguish 4 colors on the map: region with 0-patient occurrence (i.e., 0%, dark blue), regions with 1 patient occurrence (i.e., 20%, light blue), regions with 2-patient occurrence (i.e., 40%, cyan) and regions with 3-patient occurrence (i.e., 60%, yellow). No regions with 4- or 5-patient occurrence were found. Obviously, the occurrence map displays much more color shades when the number of analyzed patients increases.

Finally, the bottom-right part of the GUI can be used to apply some filters on the population, in order to select some sub-set of patients basing on: gender, familiarity, onset age, first visit year and delay between the onset age and the first visit.

## 3. Results and Discussion

### 3.1. Parameters Setup

Several options are available for the user to elaborate patient pictures with the described tool. It is worth noting that these parameters are tuned by the user for each patient to achieve optimal results, starting from the default options. A summary of the most critical aspects of the tuning is provided below, along with a discussion of the effects of the main parameters on the vitiligo detection.

Face contour: the face contour is needed to normalize each patient’s data with respect to the template face, in the statistical data elaboration. Thus, this choice has no effect in the patches’ detection. The contour can be roughly defined, only caring that the vitiligo pixels are inside the contour.

ROIs: the definition of the ROIs allows for the method adaptiveness. This has a direct effect on the patches’ definition, since the global threshold is scaled with respect to each ROI’s intensity values. The ROIs can be interactively defined, deleted, edited to achieve a good detection, depending on the particular picture. Nevertheless, ROIs do not need to precisely contour the patches (especially in the case of small disconnected patches), since the algorithm segments the ROI to discern between vitiligo pixels and healthy pixels. The aim of the ROIs is to define different face macro-regions were different intensity conditions are present.

Image filter: the tool provides four different images filtering which allows to emphasize the contrast between vitiligo and healthy pixels. The effect of each filter on the specific detection can be displayed interactively, thus the user can assess the best performing filter in real time for each patient. The performed trials demonstrated that filter 4 generally is the most effective in highlighting vitiligo patches, thus it is proposed as default setting in the tool. This can be explained by looking at Figure 4: green and blue image channels show the vitiligo patches (Figure 4b,c), while red channel shows the overall skin color (Figure 4a). This is the reason why filter 4 was defined by summing the blue and the green channel (i.e., the vitiligo contribution) and subtracting the red channel (i.e., normalization with respect to the skin color). Nevertheless, in some specific cases other filters can be more effective, thus they are available in the tool for a fine tuning.

**Figure 7:**
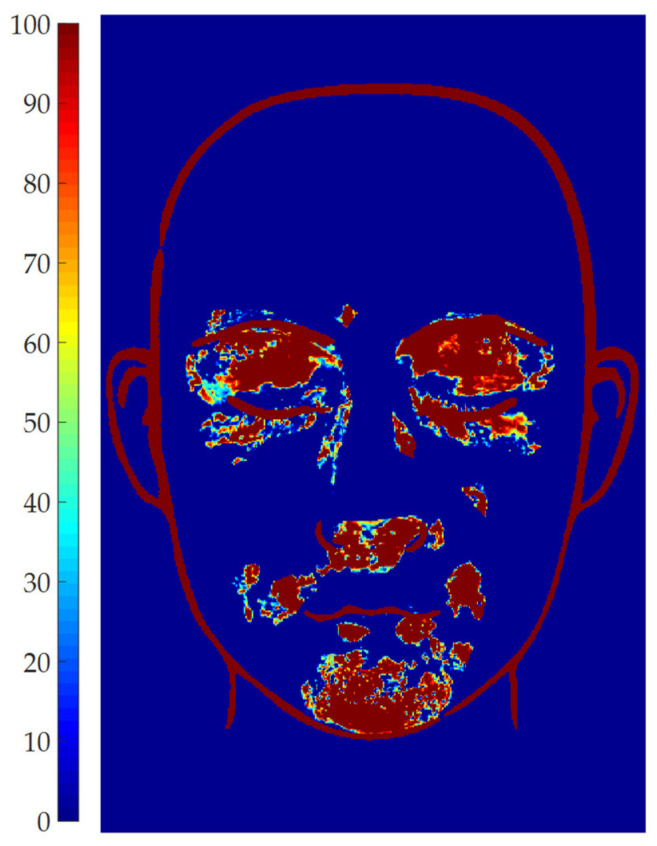
Vitiligo patches detection for different global threshold values for patient 15.

Global threshold: the global threshold is set by the user through a slider ranging from 0 to 1. The effect of the parameter is displayed in real time on the elaborated image, to assess which value is more suitable. The global threshold is used to scale the maximum gray level of each ROI, to determine the adaptive threshold to be applied in each ROI. This adaptive threshold (i.e., global threshold times the maximum gray level of each ROI) is used to binarize the image and, thus, to discern vitiligo pixels from healthy pixels. Even if the parameter can theoretically range between 0 and 1, a value of 0.5 is generally optimal, since it allows the adaptive algorithm to discern between vitiligo pixels (i.e., high intensity pixels) and healthy pixels (i.e., low intensity pixels). Thus, 0.5 is provided as default setting, and only small adjustments are required to improve the results. Since this is a critical parameter, its effect on the patches’ definition was investigated by setting all the other parameters to a constant, and then evaluating the effect of the global threshold in the range 0.35–0.65 (i.e., 0.5 ± 30%). To exemplify the results, the vitiligo pixels detected for each value of the global threshold were plotted in terms of occurrence map in Figure 7 for Patient 15. The region of the map marked with 100 represents the pixels which are detected as vitiligo for all the tested values of the threshold. As can be seen by Figure 7, most of the area of the vitiligo patches are marked with dark red color, meaning that the same regions were detected from most of the values of the global threshold, which mainly affects the patches contour. This demonstrated that the algorithm is robust with respect to this parameter in a wide range around 0.5, thus this parameter can be used to fine-tune the patches contour.

### 3.2. Patients set Elaboration

**Figure 8:**
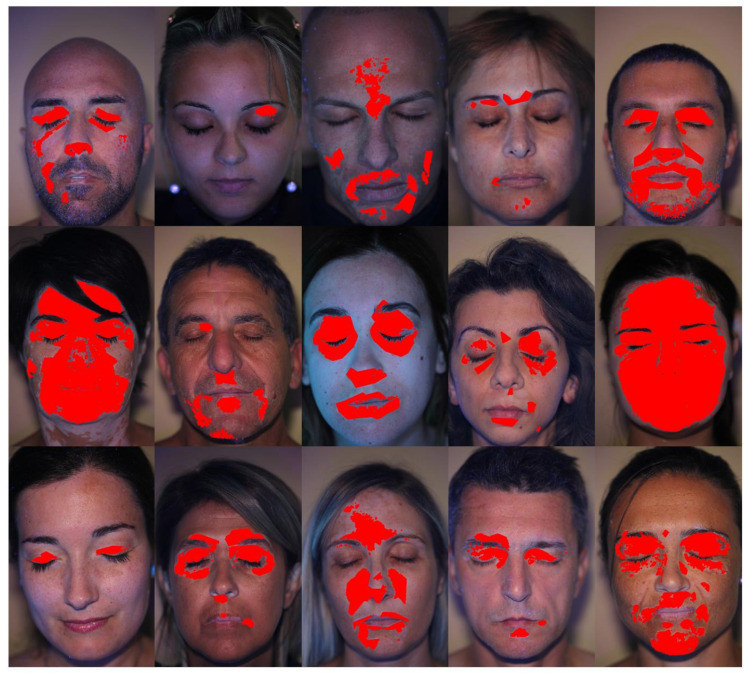
Detected vitiligo patches for the whole set of patients.

The described software was tested on a relatively small group of vitiligo patients, composed by 15 patients in an age range between 28 and 60. The number of patients is surely insufficient to draw relevant statistical conclusion about the vitiligo disease from a medical point of view. Nevertheless, the selected patients shown some peculiarities which allowed to debug the software and to test its performances with different images, having different patches contrast and different face regions involved. Figure 8 shows an overview of the detected vitiligo patches for the whole set of patients.

**Figure 9:**
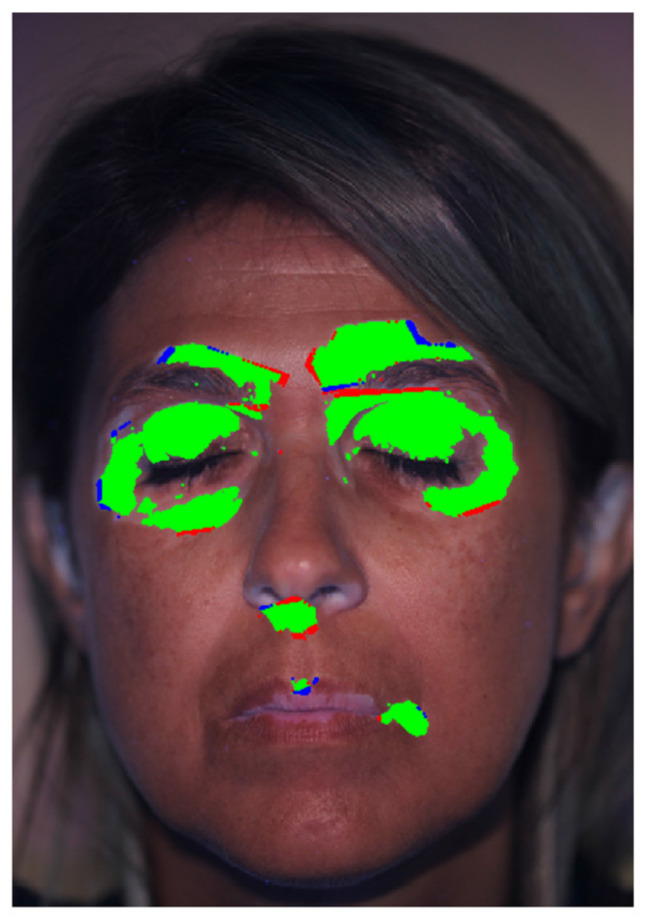
Comparison between gold standard and algorithm result for Patient 12.

The image elaboration was performed by a nurse with about 2 years of experience with vitiligo patients. The whole elaboration process took about 17 min. The elaboration was then validated by comparing the patches detected by the proposed tool and by an expert dermatologist, with a much longer experience of more than 20 years in vitiligo research. The dermatologist was asked to manually and precisely mark the vitiligo patches on the pictures, thus defining a gold standard. The comparison between the gold standard and the results obtained with the proposed tool for Patient 12 are overlapped to the patient’s picture in Figure 9: blue color represents pixels detected by the dermatologist, but not detected by the described tool (i.e., false negative), red color represents pixels detected by the described tool, but not detected by the dermatologist (i.e., false positive) and green color represents pixels detected by both the dermatologist and the described tool (i.e., successful detection). The success rate of the proposed algorithm was defined as the ratio between the green area and the total area.

**Figure 10:**
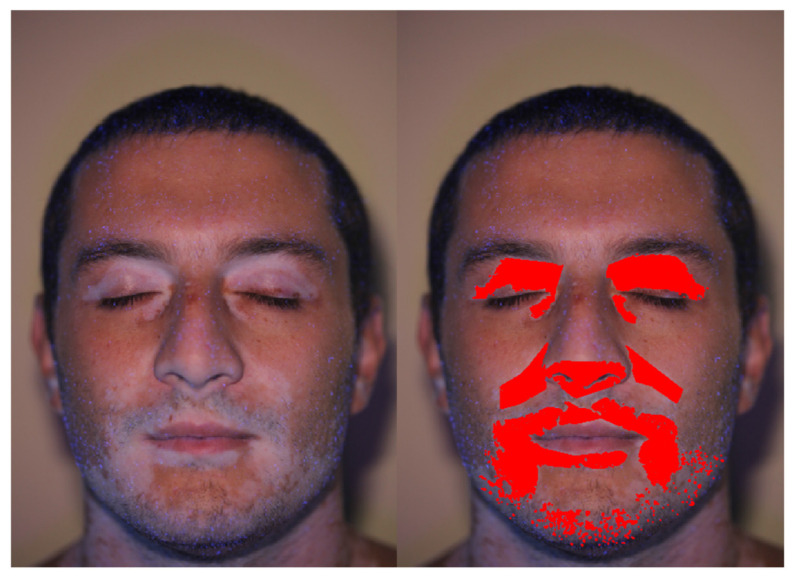
Vitiligo detection in regions with beard and non-uniform illumination.

This validation showed a success rate higher than 85% for all the studied patients, being the main differences between the dermatologist’s results and the tool results ascribed to the patches perimeter, thus confirming the patches detection and demonstrating that the developed software is a valuable tool for this kind of analysis. The elaborated patients ranged from small patched regions (such as Patients 2 and 11) to almost the whole face covered by vitiligo (such as Patients 6 and 10). In all the cases, the elaboration process was successful, only requiring adjusting the number of elaborated ROIs. It is worth noting that the proposed adaptive thresholding allowed to successfully detect vitiligo patches also in challenging conditions, as shown in Figure 10 for Patient 5. Indeed, the patient’s chin is characterized by a darker lighting and by the presence of beard. Nevertheless, vitiligo patches were properly detected in the chin region.

**Figure 11:**
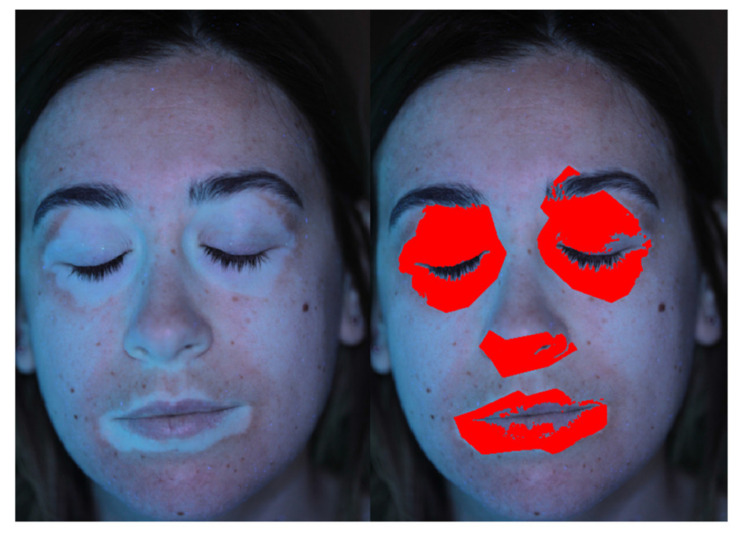
Vitiligo detection for a patient with fair skin and low patch contrast.

Another challenging elaboration example is provided by Patient 8. As shown in Figure 11, the patches contrast is low with respect to the patient’s fair skin. Also, the contrast level is significantly different between mouth, eyes and nose region, and overexposed areas on the chick can be noted. Nevertheless, the proper definition of ROIs and the tuning of the global threshold, along with the adopted image filtering (blue channel in this case) allowed to detect the patched pixels excluding the healthy skin regions.

**Figure 12:**
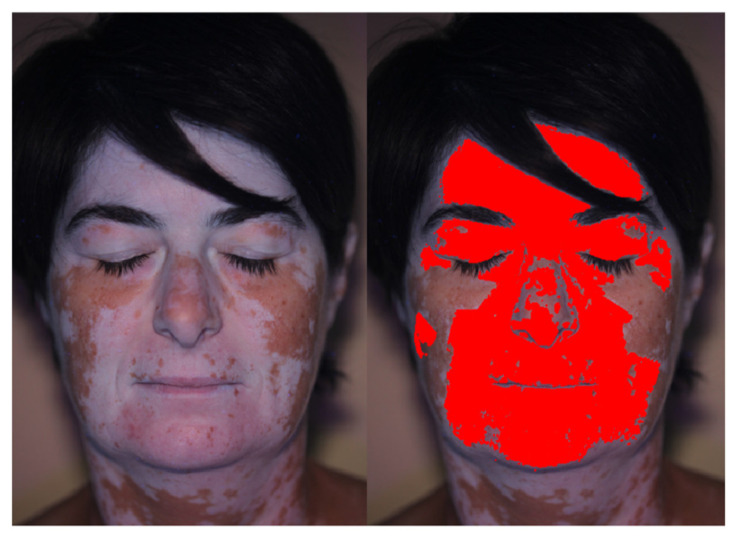
Vitiligo detection: exclusion of the neck region through ROIs definition.

Finally, Patient 6 allowed to highlight the possibility to exclude from the analysis some regions of the images. Figure 12 shows the patient’s picture, were a large portion of the neck was imaged showing evident vitiligo patches. The definition of the ROIs allowed to exclude from the analysis the neck region, which was not of interest in the present research.

**Figure 13:**
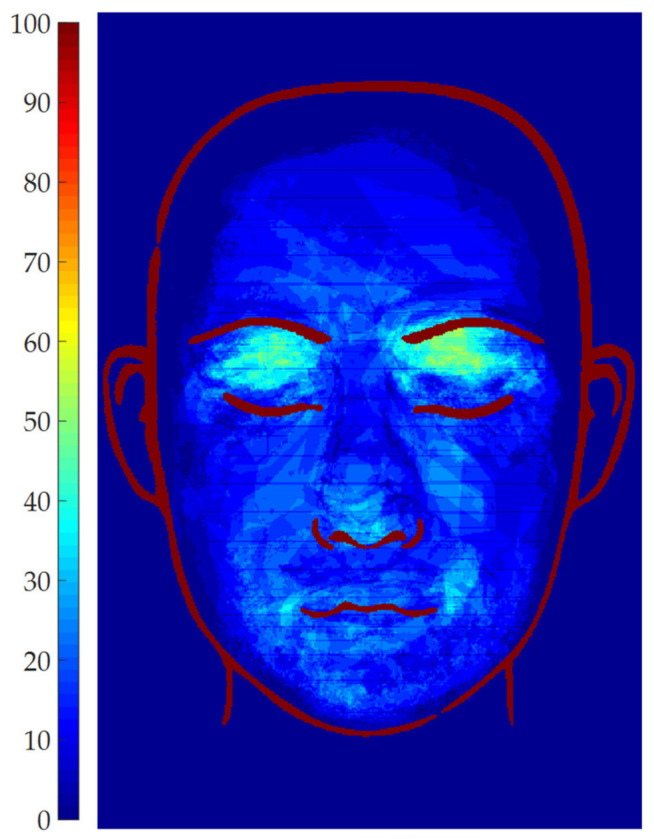
Vitiligo occurrence map for the analyzed set of patients.

Even if the number of elaborated patients was small, it was possible to analyze the data by plotting the occurrence map, in order to overlap all the patients’ patches and to assess which face regions are more subject to vitiligo in the studied population. The occurrence map for the set of patients is shown in Figure 13.

As can be noted, the normalization procedure was able to satisfactorily overlap the information coming from different patients, even if they were image at different times, with different distances from the camera, being their faces in different location of the frame and with different dimensions. Indeed, Figure 13 shows that all the patched regions are inside the face template, and also eyes, nose and mouth regions are coherently aligned with the corresponding regions of the template. Despite the small number of patients, the occurrence map already shows some trends in vitiligo patch location. Indeed, most of the patient (about 65%) show vitiligo patches on the eyelid. Also, some of them (about 45%) show vitiligo patches close to the mouth corners. All the other face regions, in the considered patient set, are less frequently affected by vitiligo.

The occurrence map of Figure 13 also allows to highlight possible issues in image acquisition and elaboration. As an example, it is possible to consider Patient 6 picture, which is shown in Figure 12. This picture shows that accidental occlusions (such as hair strands on the forehand) may influence the results, since the black hair region is not detected as vitiligo. Nevertheless, as shown in the occurrence map of Figure 13, this accidental occlusion of a single patient has a small impact on the overall map, only resulting in a slightly darker region in correspondence of the hair strand. It is worth noting that this artifact would be even more mitigated if a larger number of images was considered. Thus, even if a proper patient imaging is necessary to achieve reliable results, accidental and sporadic occlusions, which may strongly influence the single patient analysis, can be tolerated from a statistical point of view.

## 4. Conclusions

The described research activity was focused on the development of a semi-automatic tool to detect vitiligo patches by analyzing patient pictures, acquired with a black light illumination. The tool is intended for dermatologists who are not experts in image processing or in software coding. The developed graphical interface helps the user to process the picture through the needed steps: face contouring, patched ROIs definition, image filtering selection and global threshold setting. The described algorithm then performs an adaptive vitiligo detection in the selected ROIs, showing the results in real time for parameter tuning. In this preliminary paper, the tool was tested on a small set of 15 patients. Even if the population numerosity is not sufficient to draw any relevant statistical conclusion from a medical point of view, the peculiarities of the patients allowed to test the tool in various and challenging conditions, such as: picture occlusion, low contrast patches, beard and non-uniform lighting. The tool also incorporates a data processing algorithm which allows to overlap the data coming from each patient on a comprehensive occurrence map. The vitiligo patches of each patient are scaled and translated to fit a face template. A colormap is overlapped to the template: the colors of the map are proportional to the percentage of patients showing vitiligo patches in each face region. The analyzed set of data allowed to prove the effectiveness of the tool in vitiligo patch detection, allowing for the successful elaboration of all the patients and only needing about one minute per picture.

Future research will be focused on the analysis of a much larger patient set, in order to draw relevant conclusion from a medical point of view. Also, further software development can be implemented in order to extend the software to different body regions. For example, the use of machine-learning techniques could be promising in combination with the proposed approach: the described tool could be used to produce the subset of training images for the neural network and to analyze the resulting data in terms of occurrence map, while machine learning could be used to refine the results.
